# A case of well-differentiated papillary mesothelial tumor concomitant with primary gastric cancer: case report and literature review

**DOI:** 10.3389/fonc.2026.1796106

**Published:** 2026-07-22

**Authors:** Guanqing Wang, Pengyu Wang, Cheng Jiao

**Affiliations:** 1 Department of Oncology, 980 Hospital of the People’s Liberation Army Joint Logistics Support Forces, Shijiazhuang, China; 2 Department of Hepatobiliary Surgery, 980 Hospital of the People’s Liberation Army Joint Logistics Support Forces, Shijiazhuang, China; 3 Department of General Surgery, 980 Hospital of the People’s Liberation Army Joint Logistics Support Forces, Shijiazhuang, China

**Keywords:** gastric cancer (GC), immunohistochemistry (IHC), large-scale studies, mesothelioma *in situ* (MIS); Surgical treatment, well-differentiated papillary mesothelial tumor (WDPMT)

## Abstract

**Background:**

Well-differentiated papillary mesothelial tumor (WDPMT) is a rare neoplasm characterized by low malignant potential. Due to the absence of distinct clinical symptoms, WDPMT is frequently detected incidentally during surgical procedures.

**Case description:**

This report describes a patient diagnosed with primary gastric cancer (GC) and concomitant WDPMT. The lesion was identified during laparoscopic radical gastrectomy and subsequently confirmed via histopathological and immunohistochemical analysis. No recurrence or disease progression was observed during a two-year postoperative follow-up period.

**Conclusion:**

WDPMT remains a relatively rare disease. In addition to histopathological evaluation, immunohistochemistry and, where available, molecular genetic analysis contribute to diagnostic accuracy. However, conclusive evidence from large-scale studies are currently lacking. The overall prognosis of WDPMT is favorable, with surgical excision representing the standard therapeutic approach.

## Introduction

Well-differentiated papillary mesothelial tumor (WDPMT) is a rare mesothelial neoplasm characterized by low malignant potential and a low incidence, accounting for approximately 0.3% to 5% of all mesothelioma cases (1). The condition is typically asymptomatic and is often identified incidentally during abdominal or thoracic surgical procedures, or less commonly, is diagnosed following surgery in the context of complications attributable to the tumor.

Due to the limited number of cases reported worldwide, the current literature is largely confined to case reports, with a marked absence of large-scale clinical studies. In recent years, an increase in case reporting, coupled with advances in foundational research, has contributed to an improved academic understanding of WDPMT. The most recent classification published by the World Health Organization (WHO) in 2021 further refined the classification of WDPMT, now categorizing it based on tumor invasiveness and malignant potential. The present report describes a case of WDPMT occurring concomitantly with primary gastric cancer (GC), followed by a review of relevant literature.

## Case information

A 65-year-old male underwent routine health screening on November 20, 2023, during which upper gastrointestinal endoscopy revealed an ulcerative lesion measuring approximately 3.5 cm × 3.5 cm in the gastric antrum. Histopathological examination of a biopsy specimen confirmed the diagnosis of gastric adenocarcinoma. Preoperative examinations and laboratory tests showed no evidence of distant metastasis. Computed tomography (CT) demonstrated thickening of the gastric antral wall with indistinct boundaries from adjacent tissues, consistent with primary GC. The patient was subsequently hospitalized.

**Figure 1:**
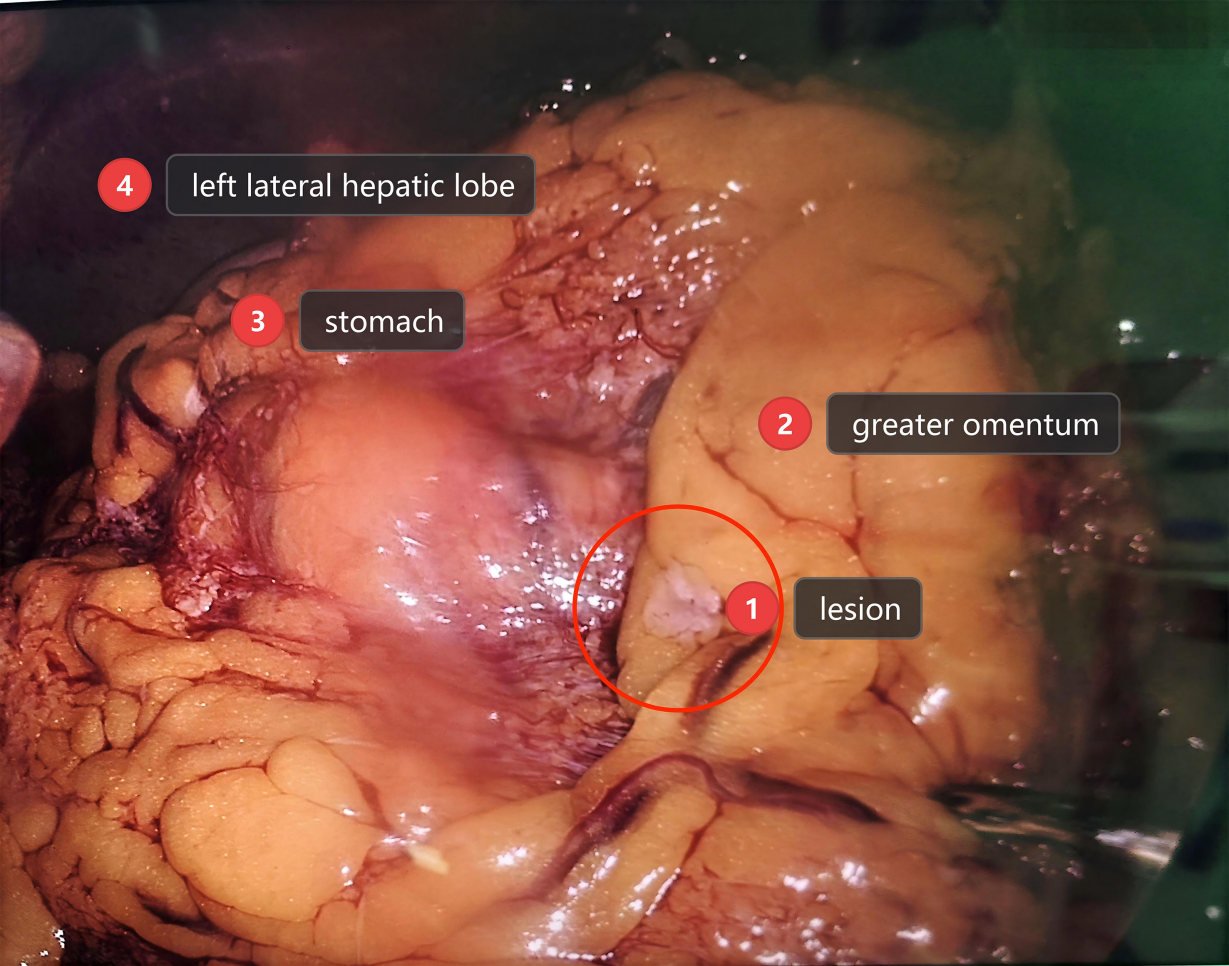
Intraoperative view showing a nodular lesion located on the greater omentum.

**Figure 2:**
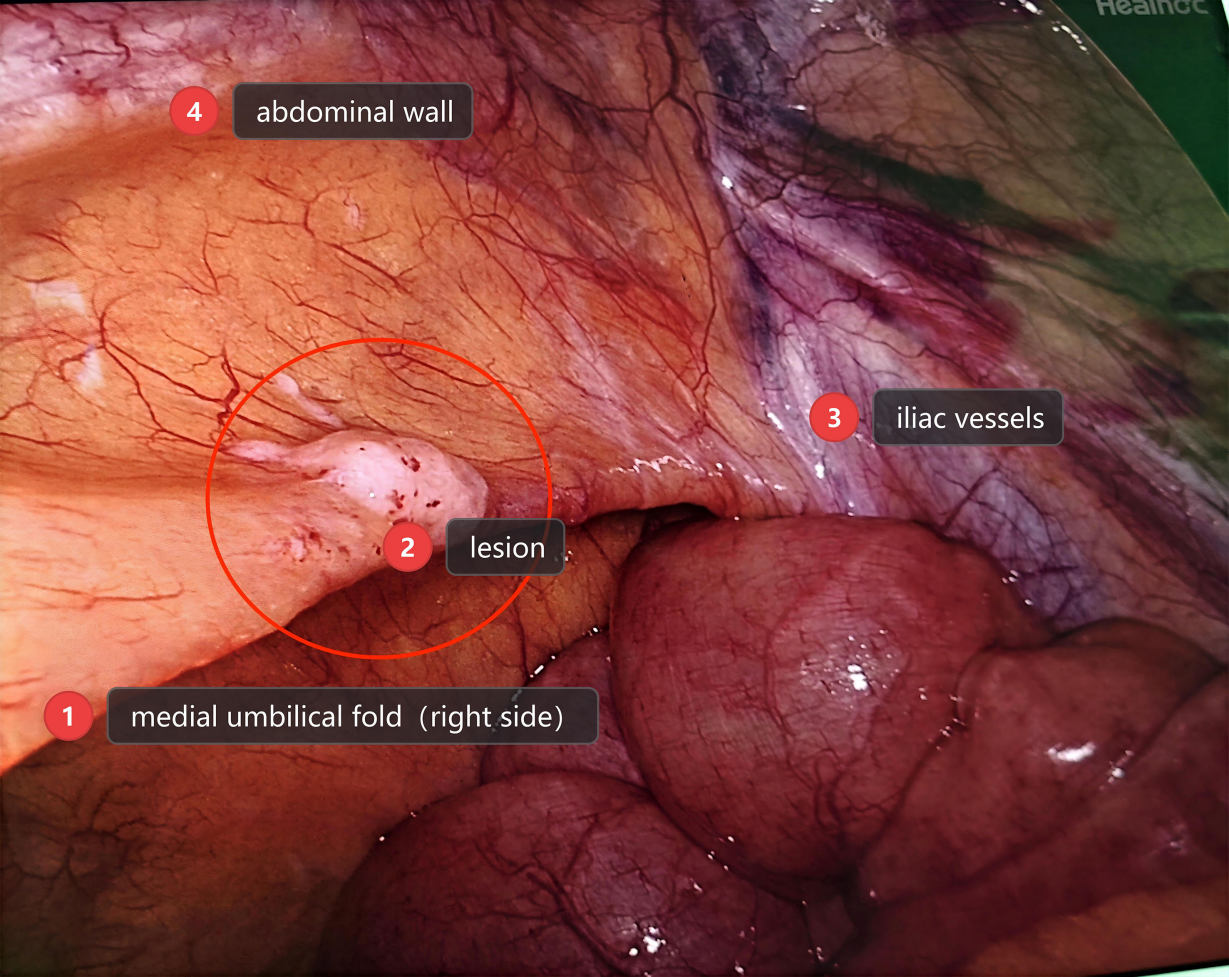
Intraoperative view depicting a nodular lesion on the peritoneal surface.

On December 5, 2023, the patient underwent laparoscopic radical distal gastrectomy. During laparoscopic exploration, multiple loosely attached soft tissue masses were found, each measuring approximately 1.0 cm in diameter, located on the greater omentum (**Figure 1**), the serosal surface of the gastric body, and the peritoneum, (**Figure 2**). No significant seroperitoneal fluid or pelvic effusion was observed. Following consultation with the patient’s family, one of the lesions was excised for intraoperative frozen section examination, which suggested a diagnosis of well-differentiated mesothelial neoplasm. The radical gastrectomy for GC was therefore continued as originally planned, and the procedure was completed with radical distal gastrectomy, followed by a Billroth I gastroduodenostomy.

**Figure 3:**
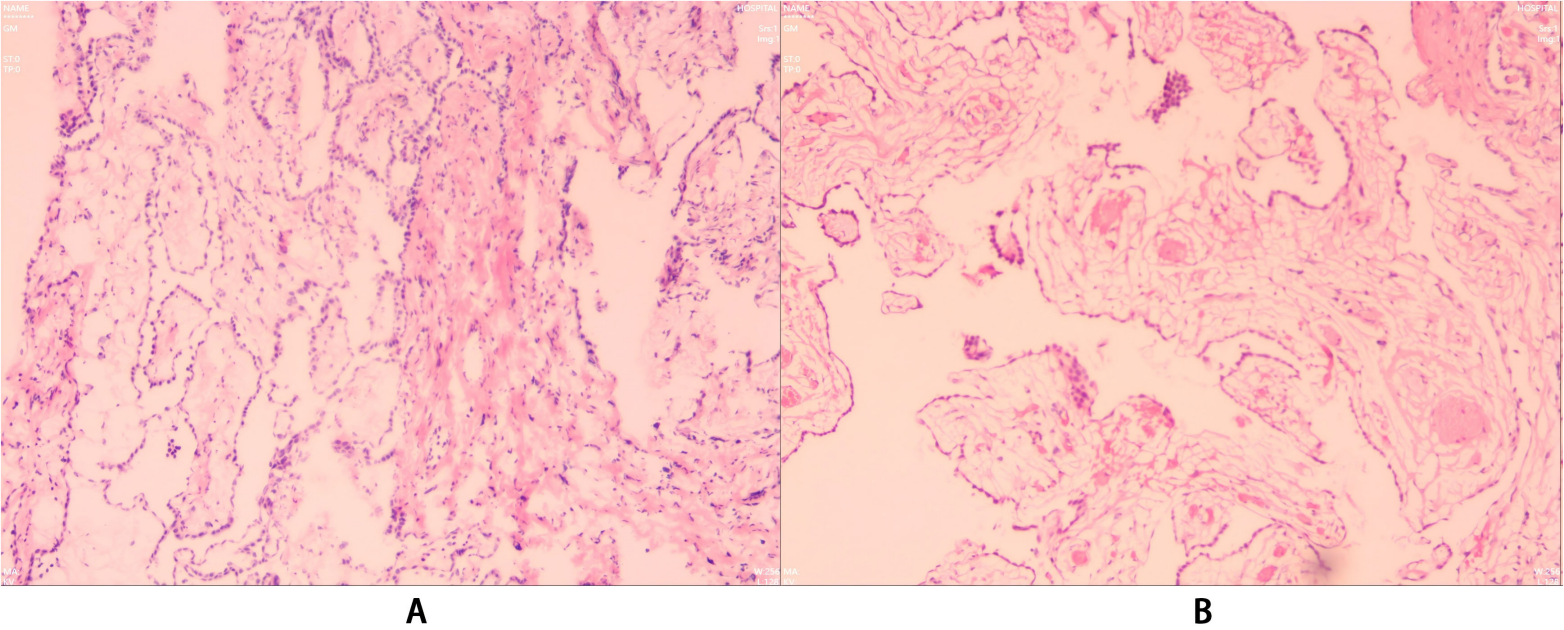
Histopathological image of the resected lesion. **(A)** Intraoperative frozen pathology of mass lesion on greater omentum; **(B)** Remnant frozen specimen of mass lesion on greater omentum observed intraoperatively. Tissue location: Remnant frozen specimen of mass lesion on greater omentum identified during operation Staining method: Paraffin section with HE staining, ×400 magnification.

Subsequently, laparoscopic resection of multiple peritoneal masses was performed. These, along with omental lesions attached to the resected gastric specimen, were submitted for pathological evaluation. Histopathological examination revealed papillary hyperplasia of mesothelial cells, consistent with a diagnosis of WDPMT (**Figure 3**). The lesion under the microscope shows multifocal papillary structures, with the surface covered by a single layer of flat mesothelial-like cells. Immunohistochemical analysis demonstrated positive staining for calretinin, cytokeratin 5/6 (CK5/6), CK7, epithelial membrane antigen (EMA), and human bone marrow endothelial cell marker-1 (HBME1), with negative staining for carcinoembryonic antigen (CEA), estrogen receptor (ER), and progesterone receptor (PR).

The postoperative course was uneventful. According to the postoperative pathological findings, the primary gastric cancer was an ulcerative-type moderately differentiated tubular adenocarcinoma (Grade II), measuring 3.5 cm × 3.5 cm × 1.0 cm, with invasion into the muscularis propria (pT2). Perineural invasion was observed, while no definite intravascular tumor thrombus was identified. Among 20 dissected perigastric lymph nodes, 1 showed metastatic involvement (pN1), and all resection margins were negative. No carcinoma was identified in the greater and lesser omental specimens. The overall pathological stage was determined as pT2N1M0 (Stage IIB, according to the 8th AJCC staging system). The patient subsequently received six cycles of intravenous adjuvant chemotherapy with the XELOX regimen (capecitabine and oxaliplatin). Follow-up assessments to date have revealed no evidence of recurrence or other significant abnormalities.

## Discussion

WDPMT represents a rare subtype of mesothelial neoplasm. Formerly known as well-differentiated papillary mesothelioma (WDPM), the terminology was updated by the World Health Organization in 2021 to better reflect the tumor’s low malignant potential. In the most recent classification of thoracic tumors published by the WHO in 2021, both the nomenclature and diagnostic criteria for WDPMT were revised. Previously designated as well-differentiated papillary mesothelioma (WDPM), the updated terminology reflects accumulating evidence that these lesions exhibit an indolent clinical course. Additionally, the 2021 WHO classification introduced a new pathological category: mesothelioma *in situ* (MIS). MIS predominantly arises in the pleura and shares histopathological features with WDPMT, yet it is now recognized as a precursor lesion to invasive mesothelioma. This indicates that, within earlier classifications, lesions diagnosed as WDPM may have included precursor entities with the potential for malignant transformation. Accordingly, the rare cases of invasive mesothelioma arising from lesions previously identified as WDPM may, in fact, have originated from undetected MIS (2).

WDPMT most frequently affects women of reproductive age but has also been reported in males and postmenopausal females. The underlying reason for this female predilection remains unclear, and no research has confirmed the role of hormones in the pathogenesis of WDPMT. This gender distribution might be related to detection bias, as women at reproductive age often undergo pelvic surgeries, leading to a higher rate of incidental tumor detection. Unlike malignant mesothelioma, no clear association has been established between WDPMT and occupational or environmental asbestos exposure (3). Specifically, peritoneal WDPMT lacks a documented link to asbestos exposure, although a few pleural cases have been reported in patients with a history of asbestos exposure. Anatomically, WDPMT can arise in various serosal locations, including the pelvic peritoneum, pleura, omentum, pericardium, tunica vaginalis, spermatic cord, uterus, ovaries, fallopian tubes, appendix, and rectal serosa. The pelvic peritoneum remains the most common site of occurrence (4).

WDPMT typically lacks characteristic clinical manifestations and is often identified incidentally during surgical procedures performed for unrelated conditions. In some instances, patients may present with non-specific symptoms such as abdominal pain or ascites. However, due to the limited number of reported cases, it remains challenging to attribute such symptoms directly to WDPMT. A limited number of reports have described severe clinical manifestations associated with WDPMT, including spontaneous bladder rupture and infertility (5, 6). In the present case, WDPMT was incidentally detected during surgery for gastric adenocarcinoma. The patient had no apparent preoperative symptoms attributable to WDPMT, and no significant ascites was observed during intraoperative exploration.

Gross examination typically reveals solitary or multiple nodular or papillary lesions, generally measuring less than 2 cm in maximum diameter. Neither macroscopic features nor imaging findings are specific to WDPMT, and definitive diagnosis relies primarily on histological examination. Microscopically, WDPMT is characterized by: (1) Growth patterns such as papillary, tubulopapillary, adenomatoid, or branching cord-like structures, with the papillary configuration being most characteristic; (2) Papillae comprising fibrovascular cores lined by a single layer of flattened to cuboidal mesothelial cells; and (3) bland cytological features, low mitotic activity, and prominent nucleoli (7). We summarized the key differential diagnostic points in **Table 1**. Laparoscopic exploration offers particular advantages in identifying WDPMT intraoperatively, especially by enabling detection of subclinical or otherwise occult lesions that may be missed during open surgical approaches. In this case, no signs related to WDPMT were found in the preoperative examination and assessment. The lesions were distributed in the peritoneum. If traditional open surgery had been adopted, there was a high probability that the WDPMT would have been missed due to insufficient exploration. This also demonstrates the significance of using laparoscopy in the timely detection and identification of WDPMT. On the one hand, laparoscopic surgery can provide a larger exploration range; on the other hand, the magnification effect facilitates the identification of smaller lesions. Timely identification and rapid pathological examination of the lesions are crucial, as they not only determine the specific type of the lesion but also whether the original surgical approach needs to be changed. For cases highly suspected of WDPMT, the priority of rapid pathological examination after lesion discovery during the operation is higher than the continuation of the original surgery. In this case, the WDPMT needed to be differentiated from metastatic lesions of gastric cancer and malignant peritoneal mesothelioma. The gross specimen of the lesion did not match the typical metastatic lesions of gastric cancer, and the rapid pathological results fully demonstrated the differences between this lesion and metastatic lesions of gastric cancer or malignant peritoneal mesothelioma.

**Table 1:** Differential diagnosis of well-differentiated papillary mesothelial tumor (WDPMT).

Feature	WDPMT	Reactive mesothelial hyperplasia (RMH)	Diffuse epithelioid mesothelioma with papillary pattern	Mesothelioma *In Situ* (MIS)
Gross appearance	Solitary or multiple nodular/papillary lesions, usually < 2 cm	Diffuse, ill-defined, no discrete mass	Diffuse peritoneal/pleural thickening or multiple masses	No grossly visible tumor (microscopic only)
Microscopic architecture	Complex arborizing papillae with fibrovascular cores; patterns: papillary, tubulopapillary, adenomatoid-like, branching cords	Simple or slender papillae, often with entrapped glands and inflammatory background	Complex papillary/tubulopapillary growth, may coexist with solid areas	Flat or cuboidal mesothelial monolayer, may morphologically mimic WDPMT
Cytological atypia	Minimal to absent; bland, uniform nuclei	Mild reactive changes, some nuclear enlargement	Moderate to marked atypia, nuclear pleomorphism	Usually minimal atypia; may appear bland
Mitotic activity	Very low (rare mitoses)	Low to moderate	Variable, often elevated	Very low or absent
Stromal invasion	Absent	Absent	Present (invasive growth)	Absent (by definition)
BAP1 IHC	Retained (nuclear positive) (BAP1 loss suggests papillary MIS)	Retained	Frequently lost (≥50% cases)	Lost (required for diagnosis)
MTAP/p16 (CDKN2A)	Retained (no homozygous deletion)	Retained	Frequently deleted (p16 loss in ~64%)	Lost (required for diagnosis)
Other IHC markers	Calretinin+, CK5/6+, WT-1+, D2-40+, L1CAM+; EMA (membranous)+/−; CEA–, BerEP4–, MOC31–, ER–, PR–	Calretinin+, CK5/6+, WT-1+; EMA (cytoplasmic, weak)+	Calretinin+, CK5/6+, WT-1+, EMA+; BAP1−; p16−	Similar to WDPMT but BAP1−/MTAP−
Molecular pathology	Lack BAP1 mutation and CDKN2A/p16 deletion	No specific genetic alterations	BAP1, CDKN2A/p16, NF2 mutations/deletions	BAP1 and/or CDKN2A loss
Clinical behavior	Indolent, low malignant potential; excellent prognosis	Self-limited, resolves after primary insult resolved	Aggressive, poor prognosis	Precursor lesion; strongly associated with eventual progression to invasive mesothelioma

Furthermore, the immunohistochemical profile of WDPMT differs from that of other malignancies and is typically characterized by positive staining for markers such as calretinin, human podoplanin (D2-40), CK5/6, CK7, EMA, and HBME1. In contrast, WDPMT generally exhibits negative staining for CEA, tumor-associated glycoprotein 72 (B72.3), epithelial cell adhesion molecule markers (BerEP4 and MOC-31), Leu-M1, ER, and PR. Cancer antigen 125 (CA125) may demonstrate partial expression. Evidence also highlights the diagnostic significance of cyclin-dependent kinase inhibitor 2A (CDKN2A/p16) status in WDPMT (8).

At the molecular level, WDPMT typically lacks the genetic alterations commonly associated with malignant mesothelioma. These include mutations or deletions in genes such as BRCA1-associated protein 1 (*BAP1*), SET domain-containing 2 (*SETD2*), neurofibromin 2 (*NF2*), cyclin-dependent kinase inhibitors 2A and 2B (*CDKN2A/B*), large tumor suppressor kinases 1 and 2 (*LATS1/2*), polybromo-1 (*PBRM1*), and *SWI/SNF*-related matrix-associated actin-dependent regulator of chromatin subfamily C member 1 (*SMARCC1*) (9). In comparison to epithelioid mesothelioma, WDPMT demonstrates higher sensitivity and specificity for paired box gene 8 (*PAX8*) staining, which may serve as an additional diagnostic marker (10). A retrospective study involving 21 patients with WDPMT indicated that loss of BAP1 expression was associated with histological features more suggestive of invasive mesothelioma. Based on these findings, it has been proposed that BAP1 immunohistochemistry (IHC) may be particularly useful in patients with multifocal WDPMT and a known history of asbestos exposure (11). However, BAP1 IHC was not yet incorporated into our departmental protocol at the time of this case.

The biological behavior of WDPMT remains incompletely understood. No standardized clinical management guidelines have been established to date and the optimal management strategy remains a subject of debate. Nevertheless, the tumor is generally considered to exhibit low malignant potential and an indolent clinical course. Surgical resection is regarded as the primary treatment modality. In asymptomatic patients, adjuvant therapy is typically not required, and long-term surveillance may be sufficient. A retrospective study involving 75 patients with WDPMT reported no evidence of recurrence or progression over a follow-up period of up to 15 years (10). Similarly, a prospective cohort study of 54 patients with WDPMT demonstrated favorable overall survival, with no tumor-related deaths observed during a median follow-up duration of 4.5 years.

In the present case, surgical intervention was performed for the management of GC. The preoperative examinations and evaluations did not reveal any manifestations related to WDPMT through imaging methods. This is consistent with the results described in most of the literature. WDPMT lacks typical clinical manifestations and also lacks characteristic imaging manifestations that are easily detectable, which is why the discovery of this disease often requires surgical intervention and cannot be predicted through preoperative imaging assessments. The minimally invasive approach facilitated comprehensive exploration of the abdominal and pelvic cavities, facilitating the identification of multiple WDPMT lesions and permitting extensive intraoperative biopsy sampling. Postoperatively, the patient received adjuvant chemotherapy for GC and was placed under routine postoperative surveillance. Currently, there is no standardized follow-up protocol established for WDPMT. The diagnostic utility of preoperative imaging modalities such as CT and ultrasonography remains limited as these techniques are generally ineffective in detecting WDPMT lesions or monitoring disease progression with sufficient sensitivity.

One case described disease progression occurring five years after the initial diagnosis of WDPMT (12). However, it remains unclear whether such events reflect true malignant transformation or represent progression from an undetected MIS lesion at the time of initial diagnosis. Due to limited long-term data, WDPMT should be managed with extended follow-up, incorporating both clinical assessment and appropriate imaging, where feasible. To date, no literature has reported a causal relationship or shared pathogenetic mechanism between WDPMT and gastric adenocarcinoma. Their synchronous occurrence in this patient is most plausibly interpreted as coincidental, reflecting the incidental detection of WDPMT during surgical exploration for gastric malignancy. This is consistent with the well-recognized clinical feature of WDPMT, which is typically asymptomatic and diagnosed incidentally during procedures performed for unrelated conditions (10, 11). Given the lack of epidemiological or molecular evidence linking these two entities, no specific screening or preventive measures for WDPMT are warranted in patients with gastric cancer beyond routine intraoperative surveillance.

## Conclusion

WDPMT is a rare mesothelial neoplasm that commonly presents without specific clinical symptoms, particularly when located within the abdominal cavity, and is often identified incidentally during surgical procedures. Histopathological examination remains essential for diagnosis, supported by IHC and, when indicated, genetic testing. While large-scale studies are lacking, current evidence suggests that the prognosis for WDPMT is generally favorable. Surgical resection remains the cornerstone of treatment.

## Data Availability

The raw data supporting the conclusions of this article will be made available by the authors, without undue reservation.
